# Dysregulation of genome-wide gene expression and DNA methylation in abnormal cloned piglets

**DOI:** 10.1186/1471-2164-15-811

**Published:** 2014-09-24

**Authors:** Guanglei Li, Qitao Jia, Jianguo Zhao, Xinyun Li, Mei Yu, Melissa S Samuel, Shuhong Zhao, Randall S Prather, Changchun Li

**Affiliations:** Key Lab of Agriculture Animal Genetics, Breeding, and Reproduction of Ministry of Education, College of Animal Science and Technology, Huazhong Agricultural University, Wuhan, 430070 People’s Republic of China; Group of Genetic modifications and establishment of biomedical models in large animals, The State Key Laboratory of Reproductive Biology, Institute of Zoology, Chinese Academy of Sciences, Beijing, 100101 People’s Republic of China; Animal Science Research Center (ASRC), Division of Animal Sciences, University of Missouri, Columbia, MO 65211 USA

**Keywords:** SCNT, Piglets, DNA methylation, Gene expression

## Abstract

**Background:**

Epigenetic modifications (especially altered DNA methylation) resulting in altered gene expression may be one reason for development failure or abnormalities in cloned animals, but the underlying mechanism of the abnormal phenotype in cloned piglets remains unknown. Some cloned piglets in our study showed abnormal phenotypes such as large tongue (longer and thicker), weak muscles, and exomphalos. Here we conducted DNA methylation (DNAm) immunoprecipitation and high throughput sequencing (MeDIP-seq) and RNA sequencing (RNA-seq) of muscle tissues of cloned piglets to investigate the relationship of abnormal DNAm with gene dysregulation and the unusual phenotypes in cloned piglets.

**Results:**

Analysis of the methylomes revealed that abnormal cloned piglets suffered more hypomethylation than hypermethylation compared to the normal cloned piglets, although the DNAm level in the CpG Island was higher in the abnormal cloned piglets. Some repetitive elements, such as SINE/tRNA-Glu Satellite/centr also showed differences. We detected 1,711 differentially expressed genes (DEGs) between the two groups, of which 243 genes also changed methylation level in the abnormal cloned piglets. The altered DNA methylation mainly affected the low and silently expressed genes. There were differences in both pathways and genes, such as the MAPK signalling pathway, the hypertrophic cardiomyopathy pathway, and the imprinted gene *PLAGL1*; all of which may play important roles in development of the abnormal phenotype.

**Conclusions:**

The abnormal cloned piglets showed substantial changes both in the DNAm and the gene expression. Our data may provide new insights into understanding the molecular mechanisms of the reprogramming of genetic information in cloned animals.

**Electronic supplementary material:**

The online version of this article (doi:10.1186/1471-2164-15-811) contains supplementary material, which is available to authorized users.

## Background

The first successfully employed nuclear transfer technology was reported by Briggs and King, who used embryonic frog blastomeres as nuclear donors in 1952 [1]. Since then, various species have been cloned [2–4]. The first cloned pigs produced by nuclear transfer from adult somatic cells were born in 2000 [5]. The cloning technology provides an opportunity to improve livestock production efficiency and to create genetic modifications in pigs for agriculture and medicine. Since the swine genome is now sequenced, the sequence information can be used to create better models of human disease [6–8]. Although the technology of SCNT has been applied successfully by many research teams, some SCNT animals have abnormal or lethal phenotypes, including facial abnormalities, pulmonary hypertension [3], contracted tendons [9], low birth weight [10, 11], as well as distinct depigmentation of the skin and hair [12]. Studies have demonstrated that Beckwith-Wiedemann syndrome (BWS), whose pathological phenotypes are similar to the abnormal phenotypes observed in our abnormal cloned piglets, is caused by epigenetic or genomic alterations in two imprinted domains on chromosome 11p15 [13]. Dysregulation of the putative imprinting center of imprinted genes (e.g., *IGF2*, *H19*, *CDKN1C*, *KCNQ1OT1*, *PLAGL1*) has been associated with BWS [14].

Based on previous studies, insufficient epigenetic reprogramming of somatic donor cells may result in phenotypic abnormalities in the offspring [15]. DNA methylation (DNAm) is believed to be the foundation for establishing and maintaining the epigenetic status in the genome [16]. DNAm occurs primarily at CpG dinucleotides (except for the regions enriched in CpG dinucleotides, which are called “CpG Islands”) in the mammalian genome [17]. In eukaryotes, DNAm results in controlling gene expression via regulation of DNA–protein interactions [18–20]. Although DNAm is one of the earliest identified epigenetic phenomena, studies of the relationship between DNAm of the whole genome and abnormal phenotypes in cloned animals (especially in cloned piglets) are lacking. Previous reports have mainly focused on cloned embryos [21–23], or detected methylation at certain repetitive regions or in individual genes [24, 25].

Methylated DNA immunoprecipitation combined with high throughput sequencing (MeDIP-seq) provides an efficient way to analyze DNAm of the entire genome [26–28]. Next-generation sequencing with RNA sequencing (RNA-seq) has shown considerable power for the analysis of gene expression without predefined transcripts or laborious cloning steps [29, 30]. Two studies analyzed the DNAm and gene expression patterns in normal or deceased cloned pigs using liquid chromatography and microarray hybridization [31, 32]. Altered DNAm and gene expression have not yet been investigated by RNA-seq and MeDIP-seq at the whole genome in cloned pigs with normal or abnormal phenotypes. MeDIP-seq and RNA-seq should provide more comprehensive evidence to understand the genome regions related to abnormalities in cloned pigs. Our work may contribute to the understanding of the epigenetic mechanisms which occur in cloned piglets with abnormal phenotypes.

## Results

### Global DNAm in cloned piglets

Biceps femoris and tongue muscles are both derived from the mesoderm and both are skeletal muscle, a form of striated muscle. Compared to other tissues, skeletal muscle consists of few cell types; thus using only biceps femoris muscle for sequencing results in a clean analysis, as there is not a mix of multiple cell types. A comparison of simple tissues will result in more stable and similar sequencing data between individuals. Muscles from different tissues have few differences in their DNA methylomes [33]. To decipher the genome-wide DNAm profiles from abnormal and normal cloned piglets, we dissected the biceps femoris muscle. Detection of genome-wide DNAm was accomplished using an Illumina HiSeq 2000. We obtained 4.8 Gb of raw data from each group (Table 1). Raw data were filtered and uniquely mapped reads were used for follow-up analyses. To obtain a genome-wide profile of MeDIP-seq reads to the reference genome, we aligned the MeDIP-seq read sequences to the porcine genome at 10 Kb length windows, and computed the read depth of each window after normalizing the read counts of each window (Additional file 1). All of the chromosomes have been mapped with the raw data, while the densities were different among these chromosomes. The density of the normalized reads mapped to the end of almost every chromosome was higher than that of other chromosome regions.

**Table 1:** **MeDIP-seq data summary for the two groups**

Group	Total reads	Mapped reads ^a^	Unique mapped reads	Reads mapping to repetitive sequences	Unique mapping rate ^b^ (%)
Abnormal cloned group	97,959,184	72,039,043	48,916,719	18,029,585	49.94
Normal cloned group	97,959,184	73,779,660	51,792,712	19,845,230	52.87

^a^Only the alignments within two mismatches were considered.

^b^Unique Mapping Rate = Unique Mapped Reads Count/Total Reads.

### Distribution of methylation data in gene elements

**Figure 1:**
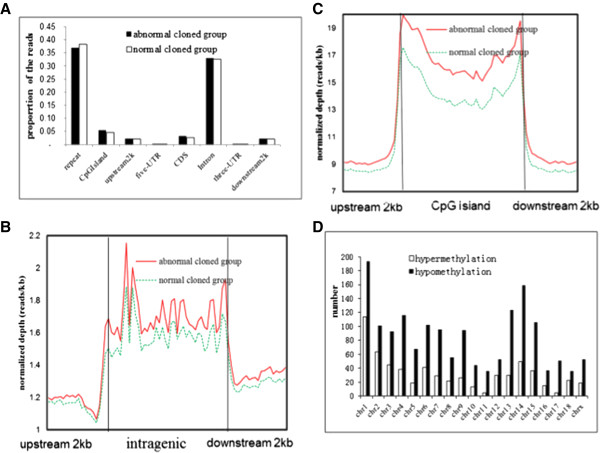
**The distribution of reads from the MeDIP-seq and analysis of the hypermethylated and hypomethylated areas. (A)** The proportion of the clean reads was inhomogeneous in the eight elements. The most two enriched elements were repetitive elements and the intron. **(B)** Normalized depth of the reads in the intragenic and the upstream 2 kb or the downstream 2 kb. No difference could be found. **(C)** The normalized depth in the CpG Island and the upstream 2 kb and downsteam 2 kb. The depth in the abnormal cloned group was more than the normal cloned group. **(D)** The number of DMRs in all of the chromosomes. The hypomethylated areas were more than the hypermethylated areas.

To further investigate the methylation data distribution within the genome, we calculated the proportion of reads in the following eight elements: repetitive elements, CpG Islands, upstream 2 kb of the transcription start sites (TSS), 5′untranslated regtions (UTRs), coding sequences (CDSs), Introns, 3′untranslated regions (UTRs), and downstream 2 kb of transcription termination sites (TTS) (Figure 1A). Repetitive elements and introns contained most of the reads. To show the variation trend of the reads in CpG Island and genebody elements in detail, we depicted the distribution line of the normalized reads (Figure 1B and C). Fewer reads in the upstream 2 kb of CpG Islands or intragenic regions were noted. However, the number of reads increased abruptly upon reaching the CpG Islands or intragenic regions, then the reads maintained a high level in the CpG islands and intragenic regions. In the downstream 2 kb of CpG Islands or intragenic regions, the reads decreased until they reached the same level as that in the upstream 2 kb. This phenomenon has been observed in other species [27, 34, 35]. Interestingly, the abnormal cloned piglets contained more normalized sequencing reads in CpG Islands and their 2 kb flanking regions than the normal cloned piglets; this phenomenon was not observed in the intragenic regions and their flanking regions.

Based on a model which is employed to specifically identify peaks (Poisson distribution, p < 0.05; Additional file 2), the coverage of the peaks in the genome was 8.28%, 8.50% in abnormal cloned piglets and normal cloned piglets respectively. We detected 146,809 peaks in the abnormal cloned piglets, 145,564 peaks in the normal cloned piglets. Although 80% of CpG sites in mammalian cells are methylated [36], the distribution of methylated CpG is not distributed equally. We checked the methylation coverage of six parts in gene components: upstream 2 kb region of TSS; 5′ UTR; CDS; introns; 3′ UTRs; and the downstream 2 kb region of TTS. In these two groups, the coverage was the highest in the 5′ UTRs, CDS and 3′ UTRs (Additional file 3). Nearly 40% of the reads were mapped to repetitive sequence regions, which contained 53 types of repetitive elements. Among these elements, seven of them were significantly different between the two groups: Satellite/centr; LINE/L1; LINE/L2; LTR/ERVL-MaLR; Simple repeat; Low complexity; and SINE/tRNA-Glu (Table 2).

**Table 2:** **Differentially methylated repetitive regions in the two groups**

Repetitive category	Reads in the abnormal cloned group	Reads in the normal cloned group	***U***-test of the reads
**Satellite/centr**	694,821^b^	652,852	24.39^a^
**LINE/L1**	4,227,144	4,718,239	-15.63
**LINE/L2**	1,790,013	1,914,343	12.78
**LTR/ERVL-MaR**	621,073	670,546	2.94
**Simple_repeat**	603,820	608,746	12.38
**Low_complexity**	296,162	296,596	6.48
**SINE/tRNA-Glu**	7,174,386	8,109,706	-53.40

^a^If |u| > 1.96, then this repetitive category displays a difference between the two groups.

^b^These reads represent the reads which were uniquely mapped to the repetitive regions of the genome.

This table contains of the differentially methylated repetitive regions in the two groups.

### Hypermethylated and hypomethylated areas in the abnormal cloned group

Next, we carried out comparisons between the two methylomes, and defined methylation in the abnormal cloned group as a hypermethylated area when the number of methylated reads mapped to the genome was higher than that in normal cloned group; or a hypomethylated area if the number of methylated reads was lower than that in the normal cloned group. Here we only considered the methylation in the regions covered by reads from both groups and DNAm in the gene elements, and the difference in read numbers was greater than two fold. Most of the hypomethylated or hypermethylated areas were detected in introns, while several were in 5′ UTRs (Table 3). There were more hypomethylated areas than hypermethylated areas in all of the chromosomes (Figure 1D). The differential methylation percentages (measured by the unique reads) in the abnormal and normal cloned piglets were 10.8% and 14.4%, respectively. The annotation of the hypermethylated and the hypomethylated areas (Additional file 4) identified 101 DMGs.

**Table 3:** **Distribution of hypermethylation and hypomethylation in each gene elements**

Category	Hypermethylation ^a^	Hypomethylation ^b^
**Upstream 2 kb**	30^c^	62
**5′UTR**	3	7
**CDS**	134	253
**Intron**	425	1219
**3′UTR**	10	21
**Downstream 2 kb**	25	64
**Total**	627	1626
**Percentage of reads**	10.8%	14.4%

^a^The number of methylated reads in the abnormal cloned group was higher than that in the normal group. The number of reads was assessed using chi-square and FDR statistical methods; p < 0.05 was considered significant.

^b^The number of methylated reads in the abnormal cloned group was lower than that in the normal cloned group. The standard evaluation was the same as for hypermethylated genes.

^c^The percentage of the reads used to calculate the hypermethylation or hypomethylation in each group was gained from the used reads dividing the whole unique reads. All of the used reads were standardized.

### Gene ontology and pathway analyses of DMGs

**Figure 2:**
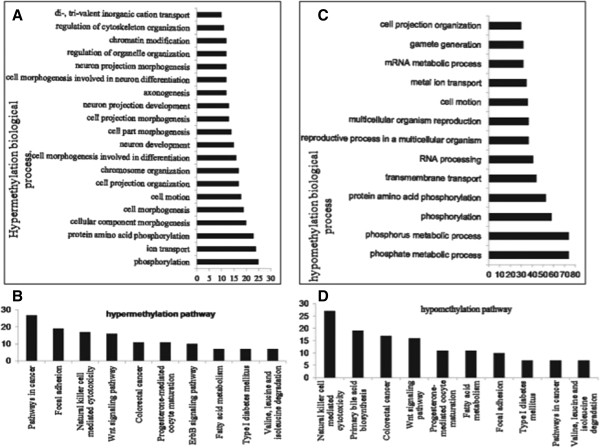
**Gene ontology and pathway analysis of the hypermethylated DMGs and hypomethylated DMGs.** DAVID software was used to conduct the analysis and partial results were shown in this figure. We only showed the prominent biological processes and pathway analysis. **(A)** Biological process of the hypermethylated DMGs. **(B)** Pathways analysis of the hypermethylated DMGs. **(C)** Biological process of the hypomethylated DMGs. **(D)** Pathways analysis of the hypomethylated DMGs.

To assess if the genes associated with differential methylation were enriched in some biological processes or pathways, we conducted gene ontology and pathway analyses using the DMGs. These DMGs were converted into human homologous genes via BioMart of Ensembl (http://asia.ensembl.org/index.html) due to the limited annotation of the pig genome. Based on Annotation, Visualization and Integrated Discovery (DAVID) analyses, 280 hypermethylated DMGs participated in 66 biological processes, and 35 terms of which reached significance (p < 0.05, Additional file 5). These biological processes mainly comprised phosphorylation; ion transport; and protein amino acid phosphorylation (Figure 2A). Also 111 hypermethylated DMGs were involved in 11 significantly different pathways, such as pathways in cancer; focal adhesion; and natural killer cell mediated cytotoxicity (Figure 2B). We also used DAVID analysis based on the hypomethylated DMGs. Among these DMGs, 720 genes participated in 127 biological processes, and 52 terms of which reached significance (p < 0.05, Additional file 6), such as phosphate metabolic process; phosphorus metabolic process; and phosphorylation (Figure 2C). Many of these processes are different from those of the hypermethylated DMGs. Two hundred sixty eight hypomethylated-DMGs participated in 24 pathways, and 13 pathways were significantly different, most of which were the same as for the hypermethylated DMGs (Figure 2D).

### Global gene expression analyses for the two groups

Previous studies have put forth the hypothesis that epigenetic alterations could induce deviations from the normal pattern of mRNA expression in the early preimplantation embryo [37]. To investigate the differences in gene expression between abnormal and normal cloned groups in the whole-genome level, we conducted RNA-seq analyses. We obtained ≈ 8.5 million unique reads in each group. Through the annotation of raw data, 11,744 transcripts were detected in the abnormal cloned group and 11,720 transcripts were detected in the normal cloned group (Additional file 7). Relative gene expression was calculated using the RPKM method (Reads per kb per million reads) to account for the variation in gene length [38]. To understand the changes in gene expression, an algorithm based on the algorithm reported by Audic et al. was developed to identify the DEGs between the two groups [39]. In total, 1,711 genes showed differential expression in the abnormal cloned group, of which 1,529 transcripts were up-regulated, and 182 were down-regulated (Table 4, Additional file 8).

**Table 4:** **Typical differentially expressed genes between the two groups**

GeneID	Normal cloned group RPKM	Abnormal cloned group RPKM	log2 Ratio Abnormal cloned group/ Normal cloned group)	Up/down	P-value	FDR	Symbol
**100153442**	0.90	65.62	6.18	Up^a^	3.21E-06	1.85E-05	*SMPDL3A*
**396959**	19.59	1046.76	5.74	Up	0	0	*CARP*
**100134978**	26.22	716.11	4.77	Up	3.44E-13	4.79E-12	*XIRP1*
**733657**	7.21	104.97	3.86	Up	1.80E-14	3.64E-13	*FBXO32*
**100048931**	5.67	72.65	3.68	Up	4.11E-14	7.71E-13	*HSPH1*
**100511413**	6.76	82.51	3.61	Up	2.10E-09	1.74E-08	*LOC100511413*
**100525306**	5.17	62.44	3.59	Up	6.08E-14	1.10E-12	*LOC100525306*
**100286778**	50.97	603.85	3.57	Up	7.68E-13	9.81E-12	*PDK4*
**100518997**	105.39	1063.03	3.33	Up	3.04E-11	3.05E-10	*FLNC*
**100337687**	53.22	526.62	3.31	Up	1.87E-13	2.90E-12	*CSRP3*
**100156435**	4728.49	522.52	-3.18	Down^b^	0	0	*TNNC1*
**100515755**	3844.93	467.28	-3.04	Down	0	0	*LOC100515755*
**396690**	7552.69	929.15	-3.02	Down	0	0	*MLC2V*
**414388**	1128.10	184.48	-2.61	Down	0	0	*TPM3*
**100513365**	53.99	12.16	-2.15	Down	5.16E-16	1.10E-14	*MGST2*

^a^This gene was up-regulated in the abnormal cloned group.

^b^This gene was down-regulated in the abnormal cloned group.

### Gene ontology and pathway analyses of the DEGs

**Figure 3:**
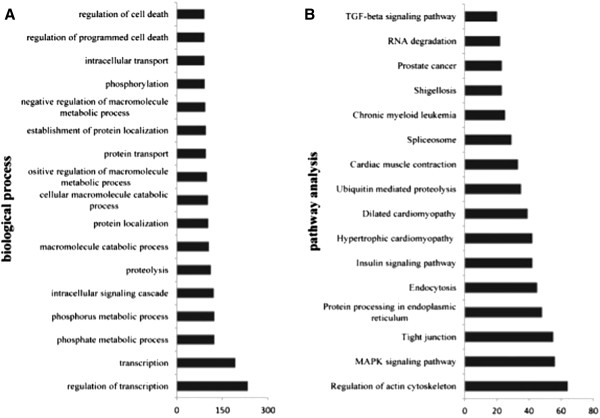
**Gene ontology and pathway analysis of the differentially expressed genes.** DAVID was employed to do the analysis by using all of the DEGs. We only showed the prominent biological processes and pathway analysis. **(A)** The biological process of the DEGs. **(B)** The pathway analysis of the DEGs.

Gene ontology analyses were conducted based on the DEGs using DAVID software. In total, 1,079 genes participated in 339 biological processes, and 243 terms reached significance (p < 0.05, Additional file 9), particularly regulation of transcription, and transcription (Figure 3A). Also, 33 pathways were identified (P < 0.05, Additional file 10), and the most prominent pathways among them were regulation of actin cytoskeleton, MAPK signalling pathway, and tight junction (Figure 3B).

### Validation of DMGs and DEGs by bisulfite sequencing and Q-PCR

**Figure 4:**
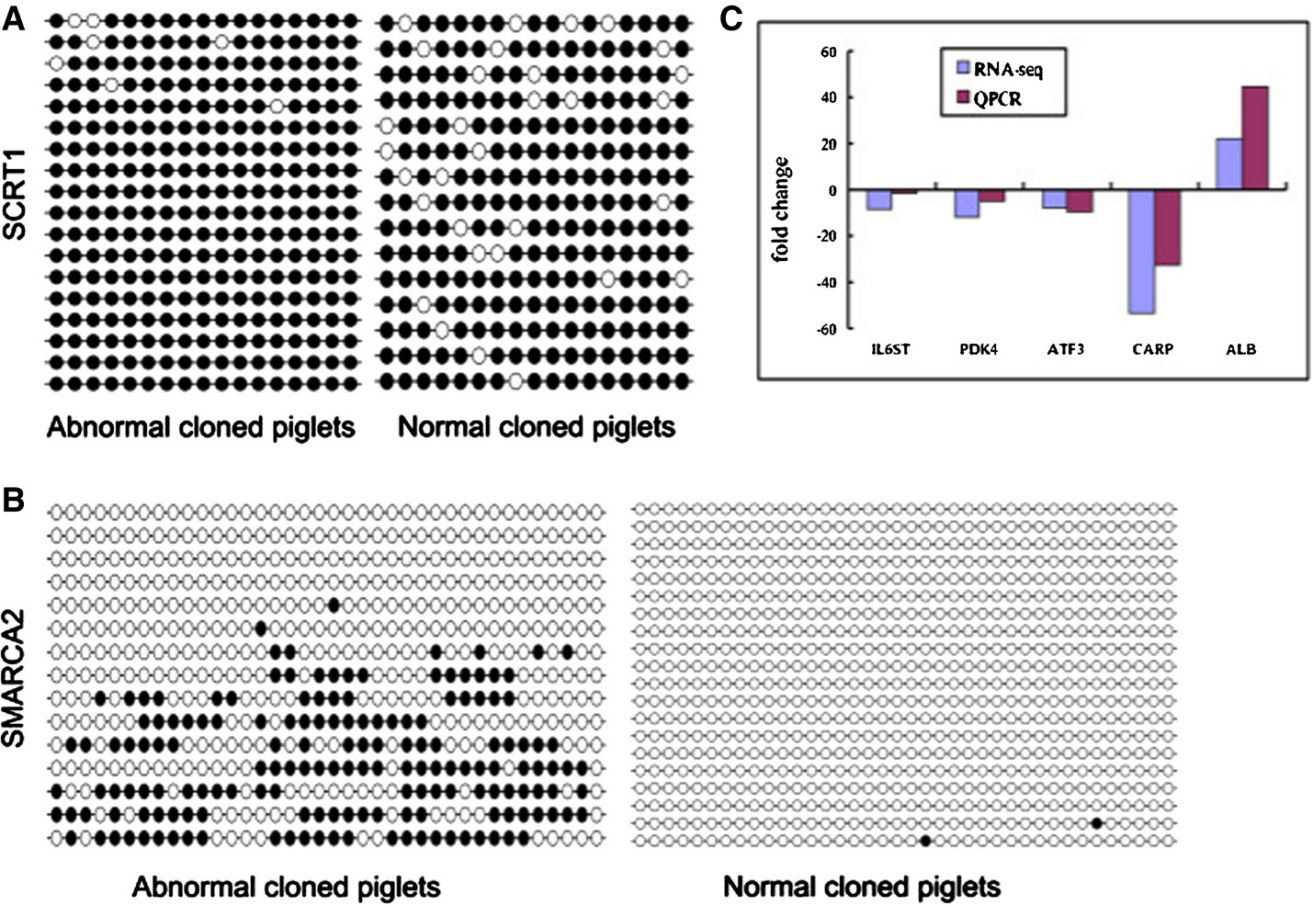
**Validation of differentially methylated genes by bisulfite sequencing and differentially expressed genes by Q-PCR. (A)** and **(B)** are the validation results of *SCRT1* and *SMARCA2* by bisulfate sequencing, respectively. The solid circles represent the methylated CpG locus and the hollow circles represent the unmethylated CpG locus. The Chi-square test showed that there are significant differences between normal and abnormal groups for each of two genes. **(C)** is the Q-PCR results of five genes. The vertical axis denotes fold change of the RNA-seq and Q-PCR in the abnormal cloned piglets compared to the normal cloned piglets. *ALB*: albumin; *ATF3*: activating transcription factor 3; *IL6ST*: interleukin-6 receptor subunit beta; *PDK4*: pyruvate dehydrogenase kinase; isozyme 4; *CARP*: cardiac ankyrin repeat protein.

To further validate the MeDIP-seq and RNA-seq data, we selected two DMGs for bisulfite sequencing and five DEGs for Q-PCR: scratch family zinc finger 1 (*SCRT1*), SWI/SNF related, matrix associated, actin dependent regulator of chromatin, subfamily a, member 2 (*SMARCA2*) (Figure 4A and B); albumin (*ALB*), activating transcription factor 3 (*ATF3*), interleukin-6 receptor subunit beta (IL6ST*),* pyruvate dehydrogenase kinase, isozyme 4 (*PDK4*), and cardiac ankyrin repeat protein (*CARP*) (Figure 4C). Bisulfite sequencing and Q-PCR results were consistent with the MeDIP-seq and RNA-seq data, indicating our sequencing data were reliable although the fold change may be a little bit different.

### The relationship of DNAm and gene expression

**Figure 5:**
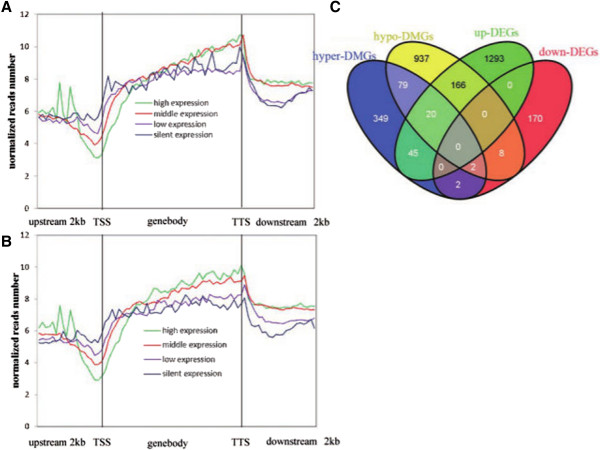
**Combined analysis of MeDIP-seq and RNA-seq. (A–B)** Distribution of MeDIP-seq reads in different expression level in the two groups. The upstream 2 kb of the TSS and downstream 2 kb of the TTS regions were divided into 20 windows, and the gene body was divided into 40 windows. The horizontal axis denotes different regions, while the vertical axis denotes the normalized read number. **(A)**: the abnormal cloned group. **(B)**: the normal cloned group. **(C)** Venn graph between the differentially expressed genes (DEGs) and the differentially methylated genes (DMGs). Each group was divided into the up-regulated and down-regulated.

DNAm has an important role in regulating gene expression and has different effects in different genetic elements. DNAm in promoter regions often suppresses gene expression, whereas DNAm in the gene body often promotes gene expression [40]. To test the relationship of DNAm and gene expression, we classified the genes into four categories according to gene expression levels: high expression (RPKM: 10–1,000), medium expression (RPKM: 1–10), low expression (RPKM: 0–1) and silent expression (RPKM: 0). We removed the silently expressed genes that were not expressed in the two groups and the genes whose RPKM values were more than 1000 in both groups. Then we plotted the distribution of the DNAm based on the four expression levels in the two groups (Figure 5A and B). In the upstream 2 kb of TSS, the methylation level remained low no matter what the gene expression levels were. It was interesting to find differential methylation around TSS, where the high expression genes had the lowest DNAm, and the silently expressed genes had the highest DNAm. In the gene body regions, there were significant differences between the two groups in the four gene expression levels. In the abnormal cloned group, the number of reads was similar (or DNAm extent) between the high expression level and the middle expression level, while it was higher than that of the low and silent expression levels. However, in the normal cloned piglet group, the order of DNAm extent from high to low was the high expression level, the middle expression level, the low expression level and the silent expression level. We can draw the conclusion that the silently expressed genes contained more DNAm than the low expression genes in the abnormal cloned group. In the downstream 2 kb of TTS, the DNAm of the four levels dropped to a low level; especially for the higher expression level. We can infer that some genes which suffered hypermethylation in the abnormal cloned group don’t express at a normal level.

Next we investigated whether the differential DNAm affected the gene expression, so we constructed a Venn diagram using the DMGs and DEGs (Figure 5C). In total, 243 genes were both differentially expressed and methylated, which accounted for 15.1% of the DMGs and 14.2% of the DEGs (Table 5, Additional file 11). The altered DNAm of these genes was primarily located in the gene body (especially in the intron regions). Through gene ontology analysis, phosphorus metabolic process and phosphate metabolic process were the significant biology processes. We also found the MAPK signalling pathway to be the most significant (Additional file 12).

**Table 5:** **DEGs potentially caused by DMGs**

GeneID	Normal cloned group RPKM	Abnormal cloned group RPKM	log2 ratio abnormal cloned group/normal cloned group)	P value	FDR	Position of regulated methylation	Symbol
**100154546**	66.2	320.5	2.28	0	0	*CDS* ^a^	*ABRA*
**100525414**	38.4	156.3	2.02	2.4E-12	2.8E-11	*Intron*	*SYNPO2*
**ENSSSCG00000016792**	44.7	139.9	1.65	4.7E-13	6.3E-12	*Intron*	-
**733596**	14.4	39.0	1.44	0	0	Intron^b^	*PLAGL1*
**100515375**	97.6	261.9	1.42	1.7E-12	2.1E-11	*Intron*	*LOC100515375*
**100153598**	96.7	238.2	1.30	1E-12	1.3E-11	*Down2k*	*TMEM110*
**ENSSSCG00000001485**	48.9	104.4	1.09	4.9E-13	6.5E-12	*CDS,Intron,*Intron	-
**100155420**	72.6	150.6	1.05	8.4E-13	1.1E-11	Intron	*NHP2L1*
**100520312**	373.4	181.8	-1.04	3.6E-39	9.2E-38	*up2k*	*LOC100520312*
**ENSSSCG00000006556**	1128.1	184.5	-2.61	0	0	*up2k Intron*	*TPM3*

^a^Some genes suffered hypomethylation in the abnormal cloned group. The font in italic means hypomethylation.

^b^Some genes suffered hypermethylation, the font in Roman means hypermethylation.

## Discussion

SCNT has considerable applications in agriculture and regenerative medicine [41–44]. Previous studies have demonstrated that abnormal phenotypes in cloned animals are caused mainly by epigenetic modifications rather than genetic mutations, as their offspring of the abnormal cloned animals tend to show normal epigenetic status and phenotypes [45, 46]. Although successful cloning has been achieved since the birth of the first cloned animal from adult somatic cells, the mechanisms related to many abnormal phenotypes or the inefficiency of SCNT technology are incompletely understood [22, 47, 48]. Few studies have focused on the gene expression and DNAm changes at an entire genome level [31, 49].

To elucidate the impact of aberrant DNAm on the abnormal development of cloned pigs, we compared the global DNAm and global gene transcript of abnormal and normal cloned group. Based on our methylome analysis, the abnormal cloned group displayed more hypomethylation than hypermethylation in the whole genome comparing to their normal litter mates, and this result is similar to other reports in pigs [21, 32, 50]. But we still cannot draw the conclusion that the abnormal cloned group was demethylated at the whole genome level, because the DNAm level in the CpG Islands were higher than in the normal cloned group (Figure 1D). Several DMGs had both hypermethylation and hypomethylation (Figure 5C). Reports have suggested that the repetitive sequences can be differentially methylated in the embryos of cows, mice and pigs [21, 23, 25, 51]. Seven differentially methylated repetitive elements were found in this study (Table 2), of which the most significant one was SINE/tRNA-Glu, this finding was also consistent with the data in another report [31]. This repetitive element in cloned mouse embryos was also reported by another group to be demethylated [52]. Satellite/centr was another aberrantly methylated repetitive element, but the methylation of this element increased in the abnormal cloned group. Satellite/centr may change expression of nearby genes via changing the binding of the transcription factors to chromatin [53]. Repetitive sequences comprise the majority of the genome and play a major architectural role to maintain the 3-dimensional structure of the nucleus [54], while the function of the repetitive elements are uncertain.

Although improper epigenetic reprogramming may lead to aberrant development of the embryo, these alterations don’t seem to explain all of the abnormal phenotypes. The aberrantly expressed genes in piglets from SCNT may also be associated with an abnormal phenotype during development; and this aberrant expression may be caused by DNAm. The RNA-seq analysis revealed numerous changes in gene expression, which were probably attributable to the physical abnormalities. These DEGs included 182 down-regulated genes and 1,529 up-regulated genes. However, many of the DEGs in the present study are different from those previously identified [31]. Different cloning procedures and embryo culture methods can cause differences in gene expression and DNAm [55], suggesting that complex mechanisms are involved in genome modifications resulting from SCNT. Through integrating profiling of DNAm and gene expression, our results revealed that the modification of the DNAm in the regions around TSS may have a direct effect on gene expression, which is accordance with previous documents [30]. In the abnormal cloned group, the altered DNAm mainly affected the low and silently expressed genes (Figure 5A- 5B). A total of 243 genes were co-altered in DNAm and gene expression, and these genes represented 14.2% of DEGs and 15.4% of DMGs. One explanation could be that only one-third of the genes in the mammalian genome are regulated by DNAm [56]. Athma reported that different levels of DNAm in the promoter region might explain as much as 12%–18% of the differences in gene expression between humans and chimpanzees [57]. A few changes in DNAm in the entire genome may not have sufficient effects on embryo development, but a few changes in DNAm in certain functional domains (e.g., core promoters) could have important effects on embryo development [58]. The survival rate of the embryos derived from SCNT is low before the first 30 days of pregnancy [59]. Here we studied the abnormal clones; and while they contained significant differences in terms of DNAm and gene expression it was not so extreme that the pregnancy was lost [60], likley these modification didn’t occur in the pluripotency markers, or other genes have compensated for gene dysregulation [60].

Imprinted genes play important roles in embryo development. But we only found a few differentially methylated or expressed genes in our data. Jiang et al. reported that there was no strong correlation between the level of the imprinted gene expression and the phenotype in pigs [10]. Among these genes, we found an imprinted gene, *PLAGL1*, which was different in both DNA methylation level and gene expression between the two groups. Expression was up-regulated in the abnormal cloned group, and the DNAm in the intron was also higher level than that of the normal cloned group. *PLAGL1* regulates growth, and it can be considered to be a tumor-suppressor gene that regulates cell-cycle arrest and apoptosis [61]. Some reports have suggested that hypomethylation of *PLAGL1* is related to BWS, which often exhibits organomegaly [62, 63]. *NNAT* is another imprinted gene which was hypomethylated in the abnormal cloned group, but we didn’t find a difference in its expression. The function of NNAT is associated with glucose transport, which may be regulated by glucokinase pathways [64]. The altered methylation of this gene may regulate other related genes. *IGF2R* is an important imprinted gene in regulating fetal growth [65], and its expression was increased in the abnormal cloned group which corresponded with our abnormal phenotype. Thus DNAm is not always associated with changes in gene expression.

The expression of *IFRD1* (also known as PC4) was up regulated in the abnormal cloned group compared to the normal group, and was hypomethylated in the gene body. *IFRD1* plays a regulatory role during the regeneration of adult muscle [66]. This gene is known to co-activate *MYOD* by promoting the transcriptional activity of *MEF2C* [67]. No studies have shown that the expression of this gene is regulated by DNAm, or that the methylation modification of *IFRD1* may serve as a novel regulator of muscle development.

The genes mentioned above showed changes both in DNA methylation and gene expression. In addition to these genes, some genes, such as *CSRP3*, *XIRP1*, and *CARP* may be related to the abnormal phenotype. All of the three genes were up regulated in the abnormal cloned piglets. *CARP* (which was validated in our Q-PCR data) is expressed mainly in the heart, lungs and muscle [68], and is induced upon injury and hypertrophy, suggesting it participates in muscle stress response pathways [69]. Xin actin-binding repeat containing 1 (*XIRP1*) is expressed in muscle satellite cells during the regeneration of skeletal muscle, and has an important role in the regulation of myoblast function [70]. Cysteine and glycine-rich protein 3 (*CSRP3*, also known as the muscle LIM protein) can promote myogenesis by activating *MYOD* [71]. *CSRP3*-knockout mice display type-I fiber atrophy and a shorter resting sarcomere length, suggesting that *CSRP3* is related to the maintenance of normal muscle characteristics [72]. These results provided a clue that most cloned animals may have some abnormal gene expression that can cause subtle phenotypic changes [55].

Gene ontology and pathway analysis are important ways to identify gene function and relationship of the DMGs or DEGs. From the results of biology process of the DMGs, there were few terms that co-exist in the hypermethylated DMGs and hypomethylated DMGs. But we also get the same terms from the pathway analysis of these two groups. It is reasonable to infer that the changed methylation of the abnormal cloned group was not random, and the genes sharing the same function may have different methylation levels. Pathway analyses of the DEGs revealed that most of the enriched genes were in the regulation of actin cytoskeleton, MAPK signalling pathway [32]. The roles of the MAPK pathway are in the proliferation, differentiation and migration of cells [32, 73, 74]. Myocyte enhancer factor 2C (*MEF2C*) in the MAPK pathway was up-regulated in abnormal cloned piglets, and its function is to maintain the differentiated phase of muscle cells. MEF2C can modulate and restrain myogenesis by activating myostatin and myostatin-dependent gene processing in pigs [75]. Filamin-C-like isoform 1 (*FLNC*) in the MAPK pathway was also expressed at high levels among these DEGs. FLNC plays a crucial part in muscle development and maintains the structural integrity of muscle [76]. Hypertrophic cardiomyopathy pathway was another significant pathway in MAPK signalling. In this pathway some genes, *MLC2V*, *MYL3* and *TPM3*, which are related to muscle development were expressed highly in the normal cloned piglets, but they were all expressed at a lower level in the abnormal cloned piglets. Abnormal muscle development was the prominent symptom in our cloned piglets, so these significantly expressed or methylated genes may be good candidates to further analyze the mechanisms of abnormal cloned pigs.

## Conclusion

Our study combined gene expression and DNAm at the entire genome level using high throughput sequencing to compare the differences and analyse the relationship of DNAm and gene expression in abnormal and normal cloned piglets. The abnormal cloned group suffered many alterations in DNA methylation and gene expression. Among them, we found some genes and pathways which may be related the abnormal phenotype. Our data may provide new insights into understanding the molecular mechanisms of the reprogramming of genetic information in cloned animals.

## Methods

### Ethics statement

All animal experimentation was conducted at the University of Missouri. All procedures were preapproved by the University of Missouri Institutional Animal Care and Use Committee (#s 3319 and 3947). Veterinary staff was consulted on a daily basis regarding the health and care of the cloned piglets.

### Cloned piglets

All of the surrogate sows were raised with standard rations and water in an experimental pig farm or in the Animal Science Research Center (ASRC) at the University of Missouri-Columbia. All of the cloned piglets produced by Somatic Cell Nuclear Transfer (SCNT) program [77] were delivered by caesarean section on Day 117 of gestation and were raised in the ASRC. Samples of biceps femoris muscles from four normal cloned piglets at Day 1 after birth and three abnormal cloned piglets at the same age were collected. All the donor cells for producing SCNT cloned piglets were from the fibroblast cells (3 abnormal and 2 normal cloned piglets from Day35 embryonic fibroblast cells from a male large white pig; Another 2 normal cloned piglets from ear fibroblast cells at day1 after birth from another male large white pig). The three abnormal cloned piglets were new-born and they had some abnormal phenotypes such as macroglossia, standing and walking disabilities, and acromphalus similar to BWS patients. All samples were flash-frozen in liquid nitrogen and stored at -80°C.

### DNA isolation and MeDIP-seq

DNA was isolated from muscle samples using the phenol-chloroform method. DNA was sonicated to gain fragments in the range 100–500 bp, and dAs were added to the 3′ ends of the extracted DNA. The adaptors were then ligated to the two ends of each fragment. Detailed steps can be referenced from the protocol of the Paired-End DNA Sample Prep kit (Illumina). The double-stranded DNA was denatured, and then an antibody that recognizes 5-methylcytosine (5-mc) was used to immunoprecipitate the DNA fragments containing regions of methylated-CpGs (Magnetic Methylated DNA Immunoprecipitation kit; Diagenode). After PCR amplification of the enriched fragments, we selected the 220–320 bp fragments using a Gel Extraction kit (28706; Qiagen). The selected DNA fragments were quantified using an Agilent 2100 Analyzer. The quantified library was sequenced using an Illumina HiSeq 2000 at the Beijing Genomics Institute (Shenzhen, China). Raw data were as FASTQ files and each read was 49 bp.

### MeDIP-seq sequence alignments and data analyses

The pig reference genome was downloaded from the Ensembl database (ftp://ftp.ensembl.org/pub/release-61/fasta/sus_scrofa/dna/). Repetitive datasets were obtained from RepeatMasker (Transposons) and Tandem Repeats Finder (Tandem repeats), downloaded from ftp://ftp.ensembl.org/pub/release-61/gtf/sus_scrofa/Sus_scrofa.Sscrofa9.61.gtf.gz. The upstream 2 kb and downstream 2 kb regions of CpG Islands (DNA length, > 200 bp; G + C content, > 50%; observed CpG/expected CpG, > 0.60), and intragenic regions were divided into 20 portions at the identical lengths. The 49 bp sequenced reads were aligned to the pig reference genome using the aligning software Mapping and Assembly with Qualities (MAQ), which allowed 2 bp mismatches. After alignment, and filtering the adapter sequences and possible contaminants, the uniquely mapped reads were used for further analyses. The unique reads were used to scan the peaks (a peak is the enrichment region where the reads was aligned to the same position in the genome) using the Model-based Analysis of ChIP-Seq (MACS1.4.0).

To identify hypermethylation and hypomethylation, we carried out differential analyses of several samples based on the peaks. Peak regions were assembled and the number of reads of each sample was calculated. The numbers of reads were assessed using chi-square and false discovery rate (FDR) statistical methods; p < 0.05 was considered significant. The differential methylation also needed to be in the regions covered by reads from both samples, and the difference in read number had to be greater than twofold. The pig genome contains widespread repetitive elements. To conduct a comparison between the two groups, we introduced the *U* test (which is suitable for the comparison of percentages). If |u| < 1.96, the repetitive sequence did not exhibit a difference, otherwise the difference was noted.

### RNA isolation and RNA-seq

Total RNA was extracted from samples of biceps femoris muscle using TRIzol Reagent (Invitrogen). The concentration and quality of RNA was confirmed using an Agilent 2100 system and agarose gel electrophoresis (AGE). Equal amounts of RNA from the tissue samples of three abnormal cloned piglets, and four normal cloned piglets were mixed into two pools. The two independent RNA pools were then used for library construction and sequencing by the Beijing Genome Institute. Briefly, after evaluation of the quality of total RNA, mRNA was enriched by using oligo(dT) magnetic beads. After adding the fragmentation buffer, mRNA was disrupted into short fragments (≈200 bp). First-strand cDNA was synthesized using a random hexamer primer using the mRNA fragments as templates. Then buffer, dNTPs, RNase H and DNA polymerase I were added to synthesize the second strand. Double-strand cDNA was purified with a QiaQuick PCR Extraction kit and washed with Ethidium Bromide buffer for end repair and addition of single adenine nucleotide. Finally, sequencing adaptors were ligated to the fragments. Fragments were purified by AGE and enriched by PCR amplification. Library products were ready for sequencing analyses via Illumina HiSeq™ 2000. Original data were transferred into sequence data by base calling (which was defined as raw data or raw reads and saved as FASTQ files).

### RNA-seq data analyses

To obtain clean reads, raw reads were filtered before data analysis. First, the adaptors were removed; second, reads in which unknown bases were > 10% were discarded; and third, low-quality reads (the percentage of low-quality bases of quality value ≤ 5 in a read were > 50%) were removed. Clean reads were mapped to reference sequences using SOAPaligner/soap2. Mismatches of no more than 2 bases were allowed in the alignment. The message abundance was calculated using the RPKM method [38]. To identify differentially expressed genes (DEGs), we developed an algorithm to be used between two samples by referring to “the significance of digital gene expression profiles” [39]. The probability of one gene being expressed equally between two samples was judged according to the p value corresponding to the differential gene expression test and FDR [78]. We used a FDR ≤ 0.001 and an absolute value of log2 ratio ≥ 1 as the threshold to judge the significance of the difference in gene expression. Functional annotation of DEGs was done by using DAVID. The hierarchical cluster analysis was conducted using MeV software ver2.0 with Euclidean distance for samples.

### Bisulfite sequencing

One microgram of the pooled DNA from the abnormal cloned group and the normal cloned group was bisulfite-treated using EZ DNA methylation-gold kit (Zymo Research). We amplified the gene fragments using the Ex Taq® Hot Start Enzyme (Takara) with the bisulfite-treated DNA as the template. Primers for the fragments of *SCRT1* were: forward 5′ –GTTTTTGTTGGGGTAGGGTAT- 3′, reverse 5′- CTTCCTTACTTCCTAAACCAACC-3′; Primers for the fragments of *SMARCA2* were forward 5′- TGGTAGGAATGTTTTTTGTGTT -3′, reverse 5′-TCCCATACTAACAATCTTCTCC -3′. All of the fragments were recovered using gel extraction kit (QIAGEN) and then cloned into the pMD™ 18-T Vector (Takara). More than 15 clones for each fragment were sent for sequencing.

### Quantitative real-time PCR (Q-PCR) validation of DEGs

Reverse transcription was conducted using SuperScript II Reverse Transcriptase (Invitrogen) according to manufacturer instructions, and cDNA was used in the Q-PCR with SYBR Green (Takara) to validate the RNA-seq. We designed the primers for the selected genes using the Primer Premier 5.0 program. The primers used are shown in Additional file 13. *GAPDH* was used as the housekeeping gene. The data from the Q-PCR was analyzed by the 2–^***ΔΔ***CT^ method [79], using the detailed procedure described previously [80].

### Availability of supporting data

All the RNA-seq data and the MeDIP-seq data from this study have been submitted to the NCBI Gene Expression Omnibus (http://www.ncbi.nlm.nih.gov/geo/) under SuperSeries accession No. GSE51477, including SubSeries accession No. GSE51282 for RNA-seq data (No. GSM1241829 for Abnormal_cloned_group - RNA-Seq and No. GSM1241830 for Normal_cloned_group - RNA-Seq) and SubSeries accession No. GSE51476 for MeDIP-seq data (No. GSM1246252 for Abnormal_cloned_group - MeDIP-Seq and No. GSM1246253 for Normal_cloned_group - MeDIP-Seq). The following link has been created to allow public review of record GSE51477: http://www.ncbi.nlm.nih.gov/geo/query/acc.cgi?token=khinewugfjelzsn&acc=GSE51477.

## Electronic supplementary material

Additional file 1: Distribution of MeDIP-Seq reads on each chromosome on the genome We scanned the genome which has been divided into 10 kb length windows using the raw data, and then computed the reads depth of each window, and then normalized the reads count of each window using this formula: RC*1,000,000/URC. RC: Reads count of the distinct 10 kb length window. URC: Unique reads count of the sample. (A) The abnormal cloned group (B) the normal cloned group. (PDF 903 KB)

Additional file 2: **Peaks obtained from the two groups Peak is the enrichment region where the reads was aligned to the same position in the genome.** (PDF 247 KB)

Additional file 3: **Coverage of peaks for each gene element The horizontal axis denotes the six gene-function elements (upstream 2 kb of TSS, 5′ UTRs, exons, introns, 3′ UTRs and downstream 2 kb of TTS).** The vertical axis denotes the coverage on the component peaks on the function elements. (A) The abnormal cloned group (B) the normal cloned group. (PDF 772 KB)

Additional file 4: **Differentially methylated areas in the comparison of the abnormal cloned group and the normal cloned group.** (XLS 376 KB)

Additional file 5: **Biological process analysis of the hypermethylated DMGs.** (XLS 20 KB)

Additional file 6: **Biological process analysis of the hypomethylated DMGs.** (XLS 22 KB)

Additional file 7: **Transcripts detected in both groups.** (PDF 241 KB)

Additional file 8: **DEGs in the comparison of the abnormal cloned piglets and the normal cloned piglets.** (XLS 318 KB)

Additional file 9: **Biological process analysis of the DEGs.** (XLS 42 KB)

Additional file 10: **Pathway analysis of the DEGs.** (XLS 22 KB)

Additional file 11: **The common genes between the DMGs and the DEGs.** (XLS 60 KB)

Additional file 12: **Gene ontology and pathway analysis of the common genes DAVID software was used to conduct this analysis.** (A) Biological process of the common genes (B) Pathway analysis of the common genes. (PDF 1 MB)

Additional file 13: **The primers used in the Q-PCR validation of the DEGs.** (PDF 128 KB)

## Abbreviations

SCNT: Somatic cell nuclear transfer
RNA-seq: RNA sequencing
MeDIP-seq: DNAm immunoprecipitation high throughput sequencing
DMG: Differentially methylated genes
TSS: Transcription termination site
5′UTR: 5′ untranslated region
CDS: Coding sequence
3′UTR: 3′ untranslated region
TTS: Transcription start site
DAVID: Database for on annotation, visualization and integrated discovery
RPKM: Reads per kb per million reads.
