# Supplementary figures and images for: Dysregulation of genome-wide gene expression and DNA methylation in abnormal cloned piglets
Source: BMC Genomics. 2014 Sep 24;15(1):811. doi: 10.1186/1471-2164-15-811 (PMC4189204; doi:10.1186/1471-2164-15-811)

**A**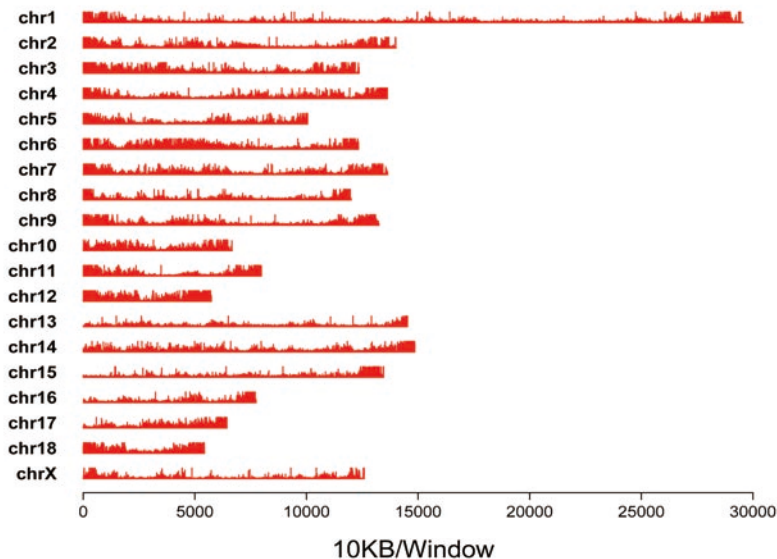**B**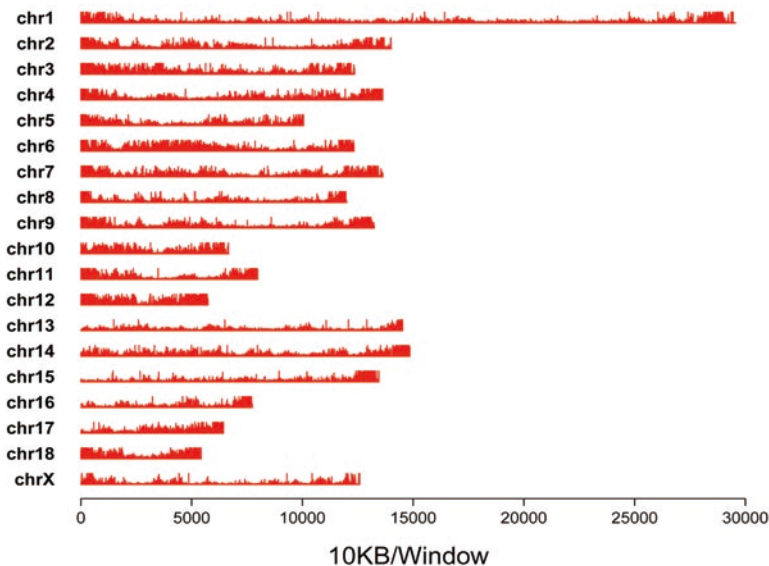

Supplement: Supplementary file 1 — Additional file 1: Distribution of MeDIP-Seq reads on each chromosome on the genome We scanned the genome which has been divided into 10 kb length windows using the raw data, and then computed the reads depth of each window, and then normalized the reads count of each window using this formula: RC*1,000,000/URC. RC: Reads count of the distinct 10 kb length window. URC: Unique reads count of the sample. (A) The abnormal cloned group (B) the normal cloned group. (PDF 903 KB) [file 12864_2013_6492_MOESM1_ESM.pdf]

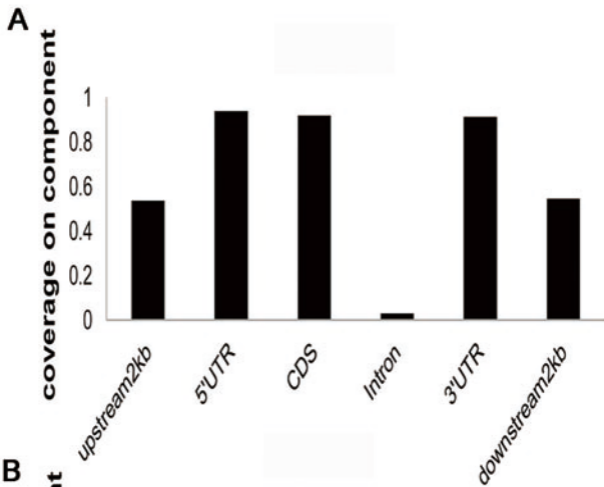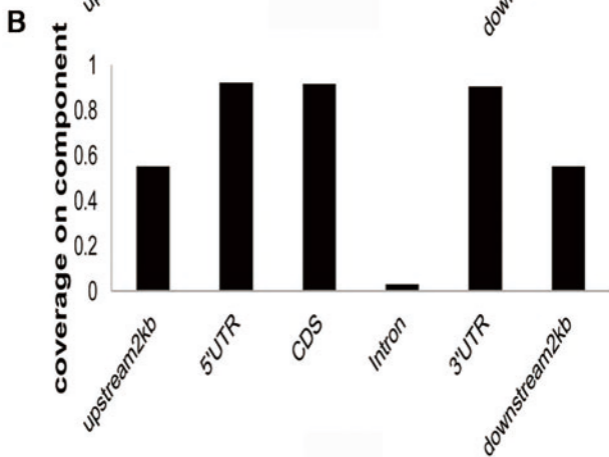

Supplement: Supplementary file 3 — Additional file 3: Coverage of peaks for each gene element The horizontal axis denotes the six gene-function elements (upstream 2 kb of TSS, 5′ UTRs, exons, introns, 3′ UTRs and downstream 2 kb of TTS). The vertical axis denotes the coverage on the component peaks on the function elements. (A) The abnormal cloned group (B) the normal cloned group. (PDF 772 KB) [file 12864_2013_6492_MOESM3_ESM.pdf]

A

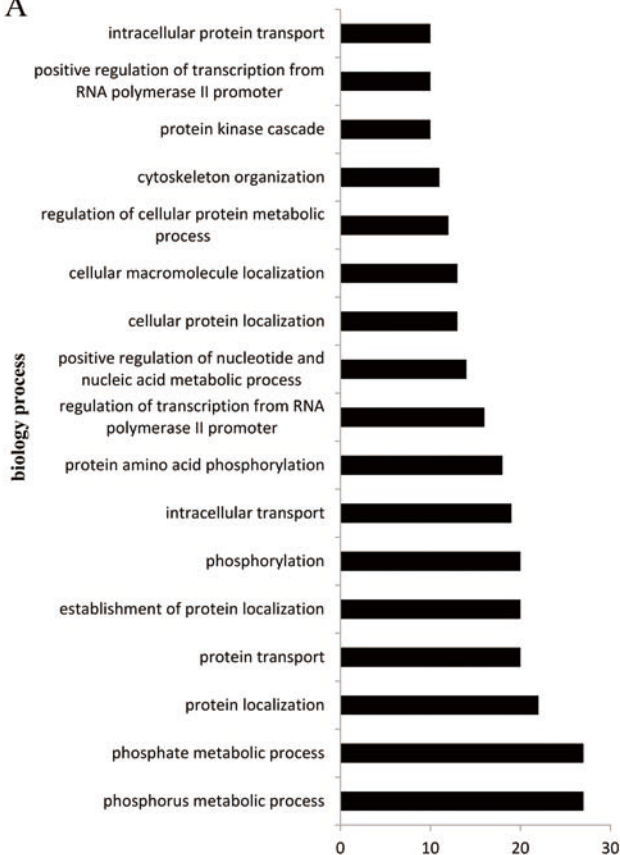

B

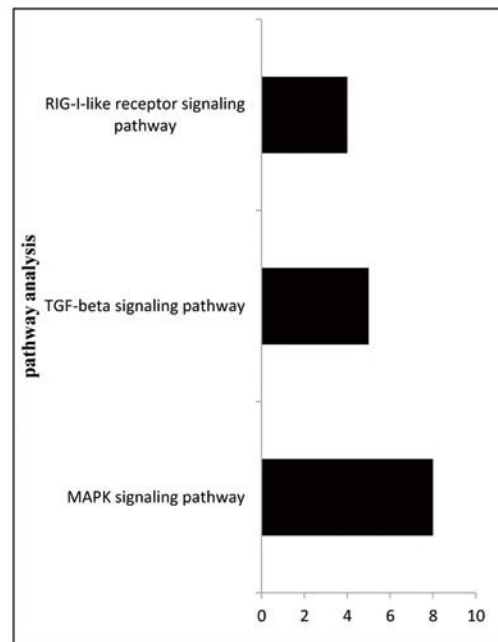

Supplement: Supplementary file 12 — Additional file 12: Gene ontology and pathway analysis of the common genes DAVID software was used to conduct this analysis. (A) Biological process of the common genes (B) Pathway analysis of the common genes. (PDF 1 MB) [file 12864_2013_6492_MOESM12_ESM.pdf]
