# Supplementary material for: Dysregulation of genome-wide gene expression and DNA methylation in abnormal cloned piglets
Source: BMC Genomics. 2014 Sep 24;15(1):811. doi: 10.1186/1471-2164-15-811 (PMC4189204; doi:10.1186/1471-2164-15-811)
Supplement: Supplementary file 2 — Additional file 2: Peaks obtained from the two groups Peak is the enrichment region where the reads was aligned to the same position in the genome. (PDF 247 KB) [file 12864_2013_6492_MOESM2_ESM.pdf]

Additional file 2： peaks obtained from two groups

	Total peaks[a]	Peak mean length	Peak Median Length	Peak Total Length	Peak Covered Size In Genome
**Abnormal cloned piglets**	146809	1276.06	1104	187337580	8.28%
**Normal cloned piglets**	145564	1321.13	1145	192308374	8.50%

[a]peak is the enrichment region where the reads was aligned to the same position in the genome
