# Supplementary material for: Dysregulation of genome-wide gene expression and DNA methylation in abnormal cloned piglets
Source: BMC Genomics. 2014 Sep 24;15(1):811. doi: 10.1186/1471-2164-15-811 (PMC4189204; doi:10.1186/1471-2164-15-811)
Supplement: Supplementary file 7 — Additional file 7: Transcripts detected in both groups. (PDF 241 KB) [file 12864_2013_6492_MOESM7_ESM.pdf]

Additional file 7： **detected transcripts in the two groups**

	Total transcripts[a]	Annotated transcript	unknown transcripts	Percentage of annotated transcripts
**Abnormal cloned piglets**	11744	7998	3746	68.1%
**Normal cloned piglets**	11720	8021	3699	68.4%

[a] the data used in this section was the clean data which was filtered as described in methods.
