# Supplementary material for: Dysregulation of genome-wide gene expression and DNA methylation in abnormal cloned piglets
Source: BMC Genomics. 2014 Sep 24;15(1):811. doi: 10.1186/1471-2164-15-811 (PMC4189204; doi:10.1186/1471-2164-15-811)
Supplement: Supplementary file 13 — Additional file 13: The primers used in the Q-PCR validation of the DEGs. (PDF 128 KB) [file 12864_2013_6492_MOESM13_ESM.pdf]

the primers for QPCR

Genes	Primer sequence (5'-3')	Tm (℃)
IL6ST[a]	Forward: TCAAAGCAAGTTTCATCAGT<br>Reverse: GCCACCTCGTCTTACAGTC	56
PDK4[b]	Forward: GGACCCTTGGGACACTT<br>Reverse: GCCAGCCATTGACTTCT	58
ATF3[c]	Forward: CACGAAAGCCGAGGTAG<br>Reverse: TATCAAATGCTGCTTCTCA	58
CARP[d]	Forward: CATTTTGAAGTTGCCGC<br>Reverse: TGGGTATGGGCAGTATCA	58
ALB[e]	Forward: TGAAGACTATCTGTCCCTGGT<br>Reverse: GTCTGCATGGAAGGTGAAG	58

[a] interleukin-6 receptor subunit beta
[b] pyruvate dehydrogenase kinase; isozyme 4
[c] activating transcription factor 3
[d] cardiac ankyrin repeat protein
[e] albumin
